# Sensitivity and specificity of the Salzburg EEG criteria for nonconvulsive status epilepticus

**DOI:** 10.1002/acn3.52184

**Published:** 2024-08-26

**Authors:** Line B. Ulvin, Kristian B. Nilsen, Erik Taubøll, Lars Etholm, Kjell Heuser

**Affiliations:** ^1^ Section for Clinical Neurophysiology, Department of Neurology Oslo University Hospital Oslo Norway; ^2^ Institute of Clinical Medicine, University of Oslo Oslo Norway; ^3^ Department of Neurology Oslo University Hospital Oslo Norway; ^4^ Section for Clinical Neurophysiology, Department of Neurosurgery Oslo University Hospital Oslo Norway

## Abstract

**Objective:**

The Salzburg EEG criteria for nonconvulsive status epilepticus (NCSE) have been proposed as consensus criteria for NCSE. We aimed to perform an independent study of their diagnostic accuracy.

**Methods:**

A prospective study was carried out at Oslo University Hospital, including all consecutive patients ≥15 years old who were referred for an EEG with an explicit or implicit question of NCSE from February 2020 to February 2022. Two independent EEG readers scored the included EEGs according to the Salzburg criteria and blinded to the clinical data. The reference standard was defined as the clinical diagnosis the patient received based on all available clinical and paraclinical data. Diagnostic accuracy in identifying “certain/possible NCSE” was assessed by calculating sensitivity, specificity, positive predictive value, and negative predictive value with their 95% confidence intervals.

**Results:**

In total, 469 patients/EEGs were included in the study. The prevalence of NCSE according to the reference standard was 11% (*n* = 53). The criteria showed a sensitivity of 94% (95% CI: 92–96%), a specificity of 77% (95% CI: 73–81%), a positive predictive value of 34% (95% CI: 30–39%), and a negative predictive value of 99% (95% CI: 98–100%). False positives for “certain NCSE” (*n* = 16) included many serial seizures and stimulus‐induced rhythmic and periodic discharges (SIRPIDs), as well as a focal cortical dysplasia. False positives for “possible NCSE” (*n* = 79) were mainly represented by different encephalopathies and postictality.

**Interpretation:**

The low specificity of the Salzburg criteria calls for refinement before implementation into daily clinical practice.

## Introduction

Nonconvulsive status epilepticus (NCSE) is defined as a state of either ongoing or repeated near‐continuous seizures without convulsions^1^ that has a potential for both short and long‐lasting consequences for the patient.^2^ Despite the wide held notion that an electroencephalogram (EEG) is crucial for the correct diagnosis and treatment of NCSE, until recently, there has been no international consensus on explicit EEG criteria for this condition. From one perspective, this is very understandable, as in most cases, an experienced EEG interpreter will have few problems recognizing ictal activity in an EEG. However, some situations can be particularly challenging and the lack of consensus criteria for NCSE may lead to variability in how medical professionals interpret EEG results, with a risk of inconsistent diagnoses.

For instance, metabolic and toxic encephalopathies can produce patterns similar to those seen in status epilepticus.^3^ Furthermore, periodic discharges constitute a controversial EEG pattern with respect to their ictal or interictal nature. Many experts now consider them to belong to an ictal‐interictal continuum (IIC), where certain characteristic features, so called “plus”‐features, are more likely to place them near the ictal end of the continuum.^3^, ^4^, ^5^ Epileptic encephalopathies often have very pathological baseline EEGs with EEG‐findings similar to NCSE. Finally, the significance of periodic or rhythmic epileptiform activity in the EEG of postanoxic patients is highly controversial.^6^, ^7^

Criteria for NCSE would be particularly useful if they could help distinguish true treatable ictal patterns from their mimickers. With the advent of more descriptive systematized EEG reading approaches,^8^ the ambition to make clear‐cut consensual EEG criteria for NCSE has made its way. In 2013 in Salzburg, during a meeting under the auspices of “The London‐Innsbruck Colloquia on status epilepticus,” an expert group developed the “Salzburg EEG criteria for nonconvulsive status epilepticus”.^9^ The criteria have since received much international attention, with calls to apply them in daily clinical practice. Leitinger et al. validated them in 2016 through a retrospective three‐center study testing the criteria on 220 patients against a reference standard, published in The Lancet Neurology. The criteria distinguish between “certain” and “possible” NCSE. Sensitivity of the criteria for “certain/possible” NCSE was 98%, specificity was 90%, positive predictive value (PPV) was 84%, and negative predictive value (NPV) was 84%. False positive patients were patients with septicemia, renal failure, hepatic failure, acute infarction, intracerebral hemorrhage, traumatic brain injury, and serial seizures.^10^

In this study, we aimed to evaluate externally the validity of the Salzburg EEG criteria through a large prospective study carried out in the most realistic setting possible. Our hypothesis was that the criteria are not specific enough to be implemented in clinical practice. A high specificity is important, as the criteria are used as a confirmatory diagnostic test in patients with symptoms.^11^ However, there is no available guideline that fixes the exact minimum specificity required in this context. We decided that we would consider 90% as the minimum required specificity. We analyzed the criteria's ability to diagnose “certain/possible NCSE” and “certain NCSE.”

## Material and Methods

We report the study using the Standards for Reporting of Diagnostic Accuracy (STARD) guidelines.^12^

### Study design and participants

We carried out a prospective diagnostic study at Oslo University Hospital (OUH) including all consecutive patients ≥15 years old who were referred for an EEG with an explicit or implicit question of NCSE from February 2020 to February 2022. All clinical neurophysiologists working in the EEG laboratory included patients/EEGs that they found relevant to the study. Patients were typically neurological or critically ill patients referred from the emergency, neurological and medical departments as well as various Intensive Care Units at the hospital.

One EEG was included per patient, in most cases a 15–30‐min standard EEG with video. Usually, the first EEG was chosen for inclusion, but in cases of sedation the EEG that was judged the most relevant was selected. In some cases, a longer 4 h EEG monitoring was included because a standard EEG had not been performed beforehand. EEGs in which technical artifacts impaired the interpretation too much were later excluded from the analysis.

### The index test

Two independent clinical neurophysiologists, LBU and LE, scored all the included EEGs strictly according to the Salzburg EEG criteria as stated in the original validation study,^10^ with the exception of the criterion related to pharmacological testing as benzodiazepines and other antiepileptic drugs are not routinely administered during standard EEGs in our hospital. All scoring was blinded to the clinical data and done at least 3 months after inclusion in the study. The EEGs were scored as either “certain NCSE,” “possible NCSE,” or “not NCSE.” The first 50 EEGs were used as pilots to adjust for differences in the understanding of the criteria between the interpreters. Differences in scoring were discussed, and a third neurophysiologist (KBN) was asked to score the recordings and included in the discussions to reduce the interrater variability. Then, the remaining EEGs were scored, and upon their completion, EEG scorings were again compared and discussed between LBU and LE. EEGs for which they could not agree were scored by KBN to reach a final decision. LBU, LE, and KBN are board‐certified clinical neurophysiologists with 8, 10, and 14 years of experience, respectively.

### The reference standard

We chose to define the reference standard as it was defined in the original validation study^10^ – as the clinical diagnosis the patient ended up with based on all available clinical and paraclinical data, including the original EEG interpretations. LBU reviewed the patients' medical records and categorized each case as “certain NCSE,” “possible NCSE,” or “not NCSE,” at least 3 months after having scored the corresponding EEG. Other clinical variables, in particular etiologies for NCSE and etiologies for those without NCSE, were also registered. Patients with “possible NCSE” as their final clinical diagnosis were excluded from the analysis due to diagnostic uncertainty.

Inevitably, the reference standard relies a lot on the original EEG interpretations made by the different clinical neurophysiologists working in the laboratory, both experienced and less experienced ones, working in a busy environment, which may lead to errors. Errors in the reference standard can cause classification bias, which may underestimate the sensitivity and the specificity of the index test. After confronting the results of the index test with the reference standard, it sometimes became evident that errors had been made in the original EEG interpretations leading to a wrong clinical diagnosis. To limit the risk of classification bias, we corrected the clinical diagnoses deemed erroneous.

### Description of EEG patterns

To help with the interpretation of results, the original EEG interpretations were sorted into categories slightly more descriptive than the Salzburg EEG criteria. These categories were “epileptiform discharges (ED) >2.5 Hz without spatiotemporal evolution,” “ED with spatiotemporal evolution regardless of frequency,” “rhythmic and periodic patterns on the IIC,” “triphasic waves on a slow background” and “other nonspecific patterns.” For rhythmic and periodic patterns on the IIC, the presence or absence of “plus” features and fluctuation was noted. “Plus” features were defined according to the 2021 version of the American Clinical Neurophysiology Society (ACNS)'s standardized critical care EEG terminology.^4^

### Statistical analyses

The sample size was determined using Buderer's formula.^13^ Based on the original validation study,^10^ we assumed a prevalence of NCSE of 30%. We chose a precision of 0.05 and expected sensitivity and specificity of at least 90% and 70% respectively. With these assumptions, the number of patients needed for the study was 461.

The interrater variability between LBU and LE was estimated using the Kappa statistic.

The diagnostic accuracy of the criteria was assessed by calculating the sensitivity, specificity, PPV, and NPV with their 95% confidence intervals. Separate analyses were made for “certain NCSE” and “certain/possible NCSE.” In the “certain/possible” analysis, EEGs that were scored as “certain” or “possible” NCSE according to the Salzburg criteria were lumped together.

Statistical significance was set at *P* < 0.05. All analyses except for the determination of the sample size were performed using SPSS (version 29) and STATA (version 17).

## Results

### Flow of participants

**Figure 1:**
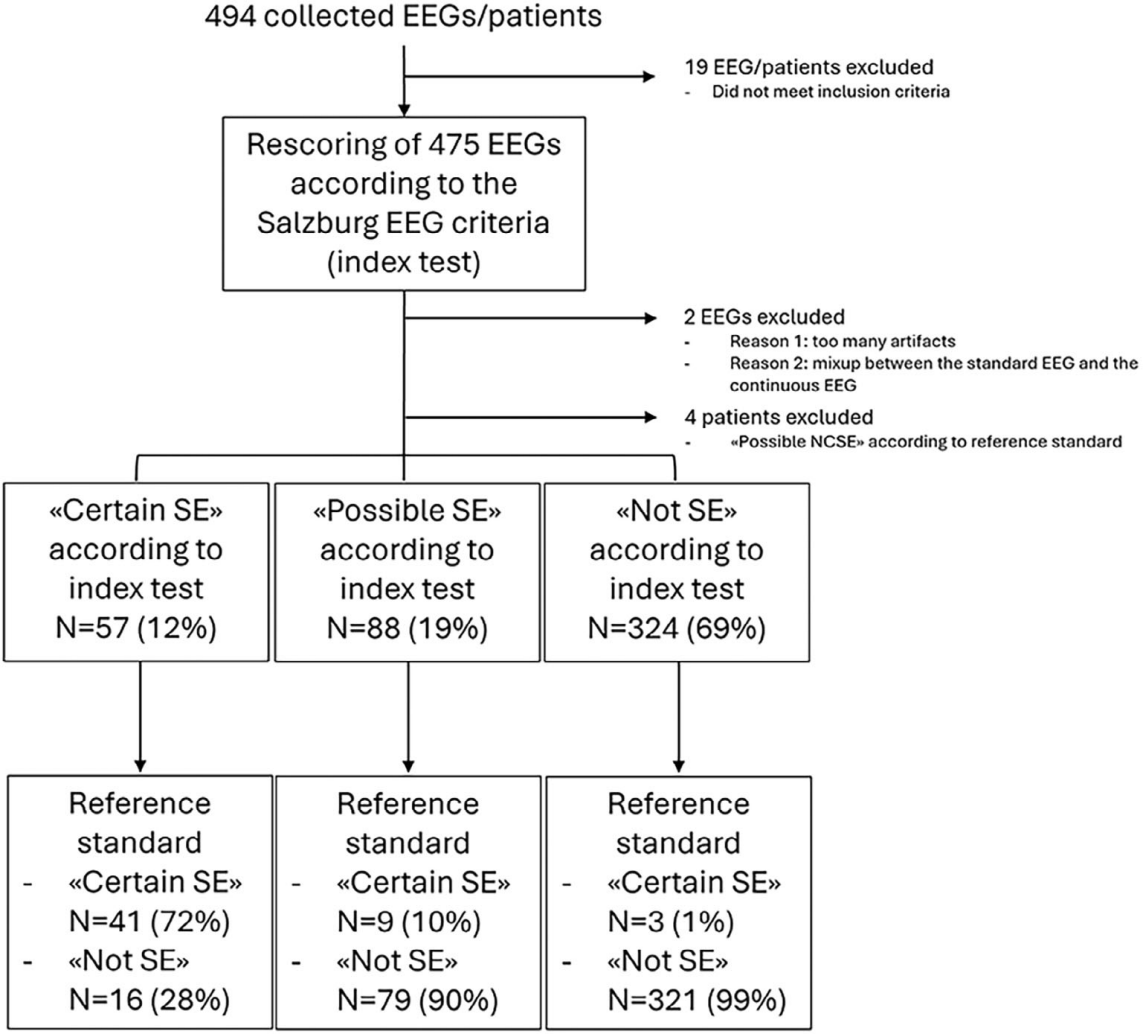
Flow diagram of participants.

In total, 494 EEGs/patients were collected, of which 469 were finally included in the study (Fig. 1). The EEGs consisted of 441 15–30 min standard EEGs and 28 4 h‐long EEG monitorings.

### Clinical characteristics of participants and reference standard

According to the reference standard, 53 of the 469 patients had “certain NCSE,” determining a prevalence of NCSE of 11%. The most frequent etiologies were intracranial tumors (19%), cerebral anoxia (16%), known epilepsy (13%), and cerebrovascular diseases (13%). The associated EEG patterns were mostly ED with spatiotemporal evolution (60%), followed by rhythmic and periodic discharges on the IIC (28%) and finally ED > 2.5 Hz without spatiotemporal evolution (8%) (Table 1).

**Table 1:** Baseline demographic and clinical characteristics of participants.

	Certain or possible NCSE according to corrected reference standard		Not NCSE according to reference standard		
	*n* = 53 (11%)		*n* = 416 (89%)		
	*n* (%)		*n* (%)		
Age range (mean; SD)	15–91 (58.6; 19.4)		15–99 (58.8; 19.3)		
Gender	Female	20 (38)	Female		162 (39)
Level of consciousness during EEG	Awake, confused or somnolent	33 (62)	Awake, confused or somnolent		245 (59)
	Stuporous or comatose	20 (38)	Stuporous or comatose		171 (41)
Known epileptic encephalopathy	Yes	0 (0)	Yes		3 (1)
Etiology	Cerebrovascular diseases	7 (13)	Serial seizures		9 (2)
	CNS infections	3 (6)	Postictal		70 (17)
	Neurodegenerative	3 (6)	PNES		3 (1)
	Intracranial tumors	10 (19)	Encephalopathy		291 (70)
	Alcohol	2 (4)	Metabolic/toxic	*n* = 32	
	Toxic	0 (0)	Sepsis	*n* = 9	
	Autoimmune disorders	3 (6)	Encephalitis/meningoencephalitis	*n* = 19	
	Mitochondriopathies	1 (2)	Cerebrovascular	*n* = 53	
	Traumatic	6 (11)	Traumatic	*n* = 32	
	Genetic	0 (0)	Neurodegenerative	*n* = 5	
	Metabolic	1 (2)	Intracranial tumors	*n* = 14	
	Cerebral anoxia	9 (16)	Cerebral anoxia	*n* = 71	
	Uncertain	1 (2)	Sedation	*n* = 18	
	Other	0 (0)	Epilepsy	*n* = 1	
	Epilepsy	7 (13)	Other	*n* = 26	
			Uncertain	*n* = 11	
			Other or uncertain		43 (10)
Etiology	Acute	25 (48)			
	Progressive	14 (26)			
	Cryptogenic	0 (0)			
	Epileptic syndrome	0 (0)			
	Chronic	14 (26)			
EEG pattern	Epileptic discharges >2.5 Hz without spatiotemporal evolution	4 (8)	Epileptic discharges >2.5 Hz without spatiotemporal evolution		1 (0)
	Epileptic discharges with spatiotemporal evolution regardless of frequency	32 (60)	Epileptic discharges with spatiotemporal evolution regardless of frequency		7 (2)
	Rhythmic and periodic discharges (IIC)	15 (28)	Rhythmic and periodic discharges (IIC)		24 (6)
	Triphasic waves on a slow background	0 (0)	Triphasic waves on a slow background		48 (11)
	Other	2 (4)	Other		336 (81)

NCSE, nonconvulsive status epilepticus; ILAE, International League Against Epilepsy; IIC, ictal‐interictal continuum; PNES, psychogenic nonepileptic seizures.

According to the reference standard, 416 of the 469 patients did not have NCSE (89%). The most frequent etiologies were encephalopathies (70%), postictality (17%), and serial seizures (2%). Among the encephalopathies, the most frequent causes were cerebral anoxia, cerebrovascular or posttraumatic encephalopathy and metabolic/toxic/septic encephalopathies. The associated EEG patterns were mostly other nonspecific patterns (81%), followed by triphasic waves on a slow background (11%), rhythmic and periodic discharges on the IIC (6%), and finally ED with spatiotemporal evolution (2%).

### Scoring of the EEGs – interrater variability

The overall interrater variability in the assessment of NCSE according to Salzburg EEG criteria was found to be Kappa = 0.55 (*P* < 0.001), 95% CI (0.48–0.61), which is considered a moderate agreement.

**Figure 2:**
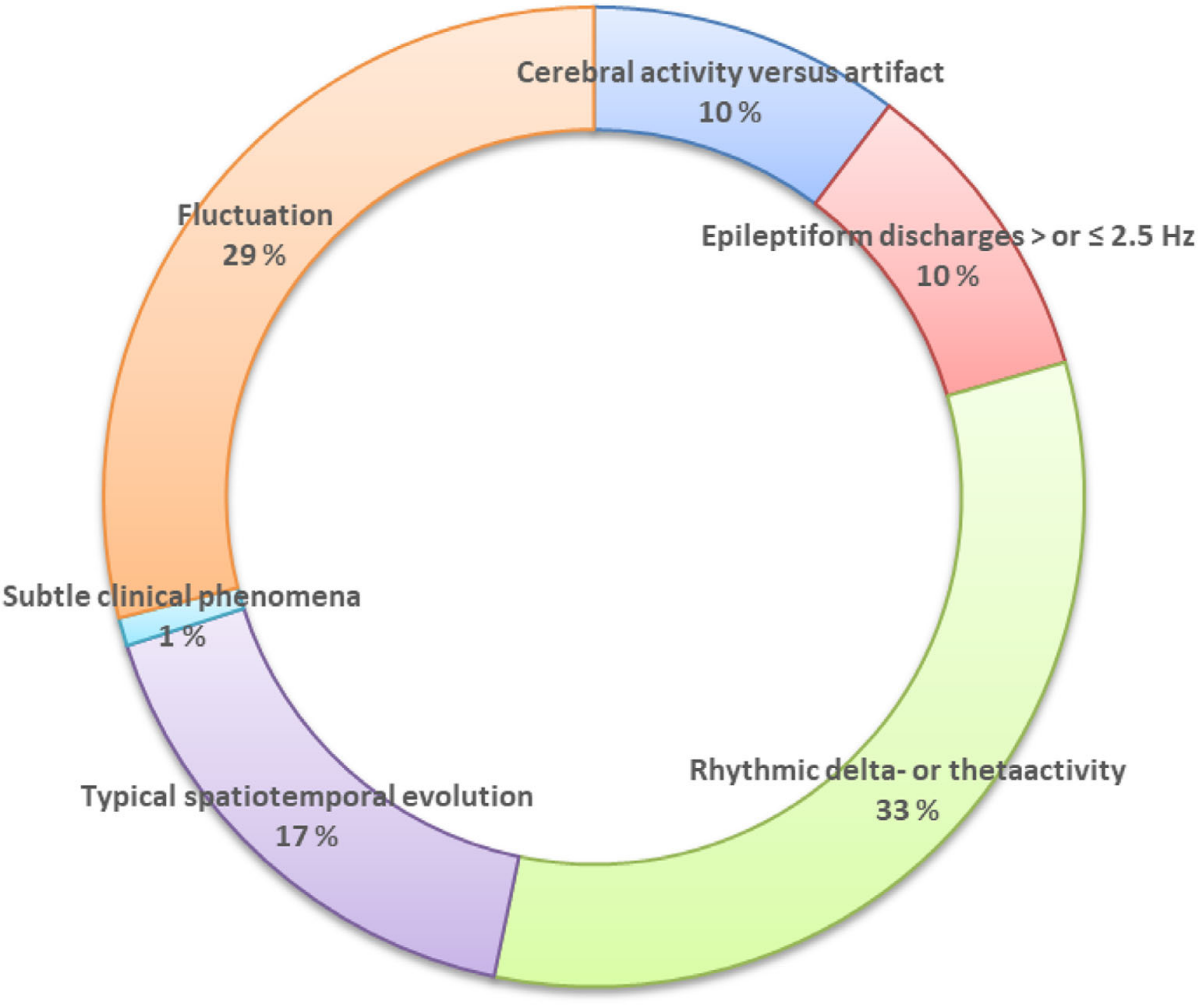
Sources of interrater variability among 107 EEGs that the two raters scored differently according to the Salzburg EEG criteria. Each sector of the diagram shows a criterion responsible for interrater disagreement and the percentage of EEGs concerned by this type of interrater disagreement: (1) distinction between cerebral activity and artifacts (in dark blue); (2) presence of epileptic discharges > or ≤2.5 Hz (in red); (3) presence of rhythmic delta‐ or theta activity (in green); (4) presence of typical spatiotemporal evolution (in purple); (4) presence of subtle clinical phenomena (in light blue); and (5) fluctuation criterion (in orange).

The sources of interrater variability are presented in Figure 2. Among the 469 EEGs, 107 were scored differently by the two raters. The most frequent sources of interrater variability concerned the criteria for rhythmic delta‐theta‐activity (RDTA) (33% of cases) and determining whether there was fluctuation of ED or fluctuation of RDTA (29%). Typically, one rater would score “fluctuating rhythmic delta activity (RDA)” leading to a “possible SE,” while the other would assess the activity as “polymorph delta activity.” The same kind of disagreement was sometimes present for ED. One rater would score “fluctuating ED,” while the other would score “sporadic/irregular ED.”

### Correcting for possible classification bias

In eight cases, subtle patterns of spatiotemporal evolution or subtle clinical phenomena had been missed by the original EEG interpreters and the corresponding patients had not received a diagnosis of NCSE. We corrected their diagnoses and treated these as certain NCSE in the reference standard.

### Diagnostic accuracy

According to the reference standard, 53 patients (11%) had certain NCSE, while 416 patients (89%) did not have NCSE. Sensitivity and specificity of the criteria in the identification of certain/possible NCSE were 94% (95% CI: 92–96%) and 77% (95% CI: 73–81%) respectively, with PPV and NPV of 34% (95% CI: 30–39%) and 99% (95% CI: 98–100%). Sensitivity and specificity of the Salzburg EEG criteria in the identification of certain NCSE were 77% (95% CI: 74–81%) and 96% (95% CI: 94–98%), respectively, with PPV and NPV of 72% (95% CI: 68–76%) and 97% (95% CI: 96–99%). Contingency tables with the number of true positives, false positives, false negatives, and true negatives are provided in Table 2.

**Table 2:** Contingency tables of the Salzburg EEG criteria against the reference standard for “certain/possible NCSE” (top table) and “certain NCSE” (bottom table).

	Certain NCSE (reference standard)	Not NCSE (reference standard)	Total
Certain or possible NCSE (Salzburg EEG criteria)	50 (TP)	95 (FP)	145
Not NCSE (Salzburg EEG criteria)	3 (FN)	321 (TN)	324
Total	53	416	469
Certain NCSE (Salzburg EEG criteria)	41 (TP)	16 (FP)	57
Not NCSE (Salzburg EEG criteria)	12 (FN)	400 (TN)	412
Total	53	416	469

NCSE, nonconvulsive status epilepticus; TP, true positive; TN, true negative; FP, false positive; FN, false negative.

We did a secondary analysis of the 28 patients with 4‐h‐long EEG monitorings. In this subgroup, six patients had certain NCSE (21%). Sensitivity and specificity of the criteria in the identification of certain/possible NCSE were 83% (95% CI: 70–97%) and 55% (95% CI: 36–73%), respectively, with PPV and NPV of 33% (95% CI: 16–51%) and 92% (95% CI: 82–100%). Sensitivity and specificity of the criteria for certain NCSE were 67% (95% CI: 49–84%) and 86% (95% CI 74–99%), respectively, with PPV and NPV of 57% (95% CI: 39–75%) and 90% (95% CI: 80–100%).

### EEG patterns and etiologies of false positive cases

**Figure 3:**
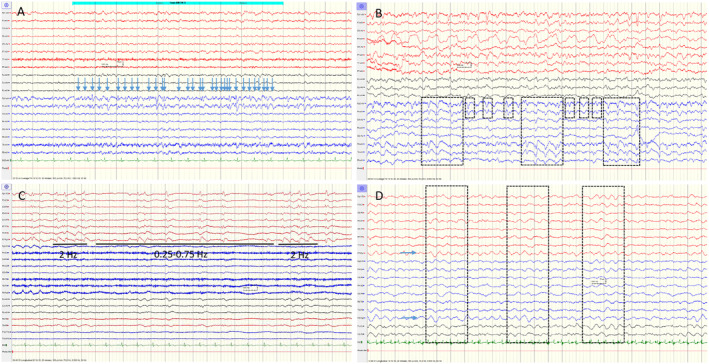
False positive EEGs for “certain” or “possible” NCSE. Vertical lines indicate 1‐sec intervals. (A) Average montage. Patient with a right frontal focal cortical dysplasia and ED > 2.5 Hz, also present in her baseline EEG (the blue arrows show 32 EDs in a 10‐sec period in electrodes Fp2 and F4). False positive for “certain” NCSE. (B) Average montage. Patient with a septic encephalopathy and triphasic waves on a slow background (the stapled boxes show fluctuation in location and morphology). False positive for “possible” NCSE based on ED < 2.5 Hz with fluctuation in location and morphology. (C) Bipolar montage. Postictality after focal seizure in a patient who gradually went back to his habitual state without any changes in AED, the EEG showing LPDs without “plus” features fluctuating between a frequency around 2 Hz (right and left sections of the tracing) and a frequency around 0.5 Hz (middle section of the tracing). False positive for “possible” NCSE based on ED < 2.5 Hz with fluctuation in frequency. (D) Bipolar montage. Patient with metabolic/septic encephalopathy and EEG showing 1.5 Hz RDA fluctuating between frontal versus diffuse location (the blue arrows show continuous RDA on channels F7‐Fp1 and F8‐Fp2, and the stapled boxes show the sections with more diffuse location). False positive for “possible” NCSE based on RDA > 0.5 Hz with fluctuation in location. NCSE, nonconvulsive status epilepticus; ED, epileptic discharges; AED, antiepileptic drugs; LPDs, lateralized periodic discharges; RDA, rhythmic delta activity.

For certain NCSE, the criteria produced 16 false positive cases. Of these, eight had serial seizures, four had encephalopathies with stimulus‐induced rhythmic, periodic, or ictal discharges (SIRPIDs), one was a case of postictality in a patient with a focal cortical dysplasia and ED > 2.5 Hz without spatiotemporal evolution but who also had a baseline EEG with the same findings (Fig. 3A). In addition, there were two cases of postanoxic encephalopathy, and one case with “uncertain” as final diagnosis, in which the clinicians leaned toward a neurodegenerative encephalopathy and in which there were ED > 2.5 Hz with fixation‐off effect.

For certain/possible NCSE, the criteria produced 95 false positive cases of which 79 were considered possible NCSE. Among these, one had serial seizures, 20 were postictal, 55 had encephalopathies (8 metabolic/toxic, 2 septic, 5 encephalitis/meningoencephalitis, 2 cerebrovascular, 6 traumatic, 1 neurodegenerative, 2 intracranial tumors, 18 postanoxic, 4 sedation and 7 other), and three were considered uncertain/other. When considering the EEG patterns of these cases, 38 were classified according to Salzburg as having ED < 2.5 Hz with fluctuation. 17 of these were originally described as having triphasic waves on a slow background (Fig. 3B), 15 as having rhythmic or periodic patterns (IIC) (Fig. 3C) and six as having other nonspecific patterns. Forty‐one cases were classified according to Salzburg as having RDTA with fluctuation. Eleven of these were originally described as having triphasic waves on a slow background and the remaining patients as having other nonspecific patterns (Fig. 3D). The patient with serial seizures did not have seizure activity on the included standard EEG but had seizure activity on a later continuous EEG recording.

### EEG patterns and etiologies of false negative cases

**Figure 4:**
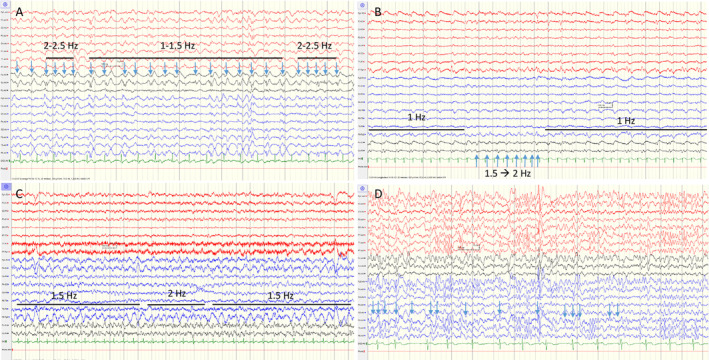
False negative EEGs for “certain” or “possible” NCSE. Vertical lines indicate 1‐sec intervals. (A) Average montage. Patient considered as a certain NCSE according to the reference standard with an EEG showing continuous 1–2.5 Hz GPDs and “plus” features (+FR). False negative for “certain NCSE,” with ED < 2.5 Hz with fluctuating frequency (channel Cz) according to the Salzburg criteria only qualifying for “possible NCSE.” (B) Bipolar montage. Patient considered as certain NCSE according to the reference standard with an EEG showing 1 Hz GPDs and “plus” features (+R), also stretches with borderline evolution. False negative for “certain NCSE,” with fluctuating ED < 2.5 Hz according to the Salzburg criteria only qualifying for “possible NCSE” (see channels Fz‐Cz and Cz‐Pz, criteria for evolution not fulfilled, as each frequency in the stretch with borderline evolution is not present for at least three cycles). (C) Bipolar montage. Patient considered as certain NCSE according to the reference standard, with a continuous rhythmic pattern in the right frontal area with superimposed spikes (lateralized RDA + S). False negative for “certain NCSE” with RDA > 0.5 Hz and fluctuating frequency in channel F8‐Fp2 only qualifying for “possible NCSE.” (D) Average montage. Patient considered as “certain NCSE” according to the reference standard, because of effect of AEDs on a later continuous EEG. False negative for “certain/possible NCSE,” with frequent sporadic ED not qualifying for NCSE according to the Salzburg criteria (see blue arrows showing the ED in channels F8 and T8). It is to be noted however that in this example, there was initially interrater variability in how to count the EDs (frequent sporadic EDs versus continuous EDs). NCSE, nonconvulsive status epilepticus; ED, epileptic discharges; AED, antiepileptic drugs; GPDs, generalized periodic discharges; RDA, rhythmic delta activity; +FR, +fast and rhythmic activity; +S, +spikes; +R, +rhythmic activity.

For certain NCSE, there were 12 false negative cases. Of these, 10 had rhythmic or periodic patterns with “plus” features and/or fluctuation (Fig. 4A–C). One was a difficult case concerning the distinction between a NCSE and an encephalopathy and on deciding on which potentials to count as ED (Fig. 4D). Depending on how you counted the potentials, you could either end up with ED > 2.5 Hz or just frequent sporadic ED. This patient received a diagnosis of NCSE according to the reference standard based on clinical and EEG‐effect of antiepileptic drug administration on a later continuous EEG recording. Finally, one was a case with repeated motor events with a negative EEG but in which the clinician decided on a status epilepticus diagnosis.

For certain/possible NCSE, there were three false negative cases, which were a patient with a periodic pattern with “plus” features that did not fulfill criteria for fluctuation as well as the two previously cited patients: the patient with a negative EEG overruled by the clinician and the patient in which it was difficult to separate a NCSE from an encephalopathy.

## Discussion

In this study, we set out to test the validity of the Salzburg EEG criteria for NCSE in an external independent manner, in the most realistic setting possible, true to daily clinical practice. With a 77% specificity (95% CI: 73–81%) and a 94% sensitivity (95% CI: 92–96%), the results confirmed our main hypothesis that the criteria for certain/possible NCSE are not specific enough to support their systematic implementation in clinical practice.

While the initial validation study performed by Leitinger et al. 2016 found a specificity of 90%, later retrospective external studies have found specificities of 89%^14^ and 60%.^15^ One retrospective study has surprisingly found a specificity of 100%.^16^ The risk of a low specificity is that many patients will receive an incorrect diagnosis of possible NCSE, which may result in unnecessary therapeutic escalation including possible anesthetic treatment, and may lead to potential complications. Receiving an incorrect diagnosis of NCSE will also shift the focus away from what is actually the problem with these patients, which may also lead to lost therapeutic opportunities. Looking at the results for the Salzburg EEG criteria in identifying only certain NCSE, specificity was markedly better, of 96% (95% CI: 94–98%). However, this was at the expense of an unacceptably low sensitivity of only 77% (95% CI: 74–81%). This is in line with the results of the initial validation study,^10^ which produced a sensitivity of 79% and a specificity of 97% when evaluating certain NCSE only, and of the study by Othman et al, which produced a sensitivity of 71% and a specificity of 100% for certain NCSE only.

Which patients have the highest risk of receiving an incorrect diagnosis of NCSE using the Salzburg EEG criteria?

Patients that were false positives for “possible NCSE” (*n* = 79) most frequently had various types of encephalopathies as etiology: postanoxic encephalopathies, followed by metabolic/toxic/septic encephalopathies, traumatic encephalopathies, and encephalitis/meningoencephalitis. The second most frequent etiology was postictality. These patients either had ED < 2.5 Hz or RDA with fluctuation according to the criteria. Looking at the original EEG descriptions of these patients, 49% had been described as “triphasic waves on a slow background,” 19% had been described as “rhythmic or periodic discharges on the IIC” and the remaining 32% had been described as “other nonspecific patterns.” A weakness of the Salzburg EEG criteria is that they do not mention how to handle triphasic waves (also known as generalized periodic discharges with triphasic morphology according to new ACNS terminology^4^). Triphasic waves have classically been associated with metabolic, toxic encephalopathies and similar. They may be difficult to separate from epileptic sharp‐and‐slow waves, and a commonly faced challenge in clinical practice is to separate status triphasicus from status epilepticus.^17^ Electrographic characteristics that can help distinguish between the two include long runs of 1–2.5 Hz triphasic waves bilaterally and symmetrically, maximal in frontocentral or posterior head regions with stimulation‐induced activation and recurrent spontaneous or low‐dose benzodiazepine‐induced attenuation.^17^ Moreover, another weakness of the criteria is the lack of a precise description of the IIC, replaced by the term “possible NCSE.” In our opinion, in addition to “fluctuation,” “plus” features are important to consider when placing a pattern on the IIC, especially if the term “possible NCSE” is to be used. Finally, RDA with fluctuation was a great contributor to false positive patients in our study and should probably, in the absence of other “plus”‐features, be considered as a nonspecific pattern.

Patients that were false positives for “certain NCSE” (*n* = 16) most frequently had received a diagnosis of serial seizures (*n* = 8), followed by SIRPIDs (*n* = 4). Whether or not these patients should have been considered as true positives may be subject for discussion, but a weakness of the criteria is that they do not quantify how much seizure activity is necessary to qualify as NCSE. For example, to amend for this, the ACNS have recently set a threshold of 10 min or 20% of a 60‐min period of recording as the minimum duration of seizure activity to qualify as NCSE.^4^ In addition, one of the false positive patients was a patient with a focal cortical dysplasia and ED > 2.5 Hz without spatiotemporal evolution but who also had a baseline EEG with the same findings. The ED were frequent, but irregular, like is often the case in focal cortical dysplasias. They lacked the rhythmicity that usually characterizes ictal patterns.^18^ We suggest that criteria for NCSE should give more importance to rhythmicity as a characteristic of ictal patterns.

Some patients that received a diagnosis of certain NCSE according to the reference standard did not receive the diagnosis using the Salzburg EEG criteria. Patients that were false negatives for “certain NCSE” (*n* = 12) were mostly patients with rhythmic or periodic discharges on the IIC with both “plus” features and fluctuation (*n* = 10). In all but one case, these patients received a diagnosis of possible NCSE according to the Salzburg criteria because they fulfilled the fluctuation criterion. The patient that did not receive a possible NCSE diagnosis was a patient with more static periodic discharges and “plus”‐features. It is important to note that patients with static rhythmic patterns, similar to Treiman's classic “continuous rhythmic pattern” (usually considered as a certain seizure pattern),^19^ may be missed by the Salzburg criteria. Depending on how conservatively the EEG‐interpreter scores fluctuation, there is a risk of a significant number of false negatives of this type.

The high number of false positive patients for “possible NCSE” largely accounts for the criteria's lack of specificity. It might be argued that “possible NCSE” merely indicates that an EEG alone cannot fully characterize the condition and that a closer clinical assessment is needed. This would suggest that the sensitivity of the criteria for diagnosing certain/possible NCSE is more critical than their specificity. However, we believe that most clinicians use the EEG as a confirmatory diagnostic test,^11^ and that one should not underestimate the power of an EEG result. An EEG result of “possible NCSE” should increase the post‐test probability of NCSE significantly. Given that clinical signs of NCSE, aside from disturbed consciousness, are often subtle, nonspecific, or even absent, clinicians often rely heavily on EEG.^10^ While paraclinical data such as cerebral imaging or laboratory results can provide alternative explanations for a disturbed level of consciousness, like acute structural lesions or metabolic derangements, these conditions are frequently proepileptogenic and may be complicated by NCSE. Thus, we consider it important for the criteria to achieve a high level of specificity in addition to a high sensitivity. In our study, we think that many of the EEG patterns that were categorized according to the Salzburg criteria as “possible NCSE” were too unspecific to be categorized as such: especially patterns of fluctuating rhythmic delta activity or fluctuating triphasic waves. For the remaining patterns, the more descriptive term “IIC” may be more adequate than “possible NCSE,” especially as recommendations on how to manage patients with these patterns are emerging with typically a less aggressive treatment approach.^4^, ^20^

Another important result of this study was the considerable interrater variability in the assessment of NCSE according to the Salzburg EEG criteria, and this despite having discussed the first 50 EEGs as pilots to make sure that interpreters had a common understanding of the criteria. Problems that we identified during the pilot were how difficult it could be to differentiate RDA with fluctuation from polymorphic delta activity and to separate ED < 2.5 Hz with fluctuation from frequent irregular sporadic ED. Other problems concerned the scoring of triphasic waves, artifacts, and changes in vigilance that mimicked spatiotemporal fluctuation and evolution.

Despite having discussed potential problems related to the application of the criteria before scoring the majority of the EEGs, the agreement between the two raters was only moderate with criteria for RDTA (33%) and criteria for fluctuation (29%) as the two major sources of disagreement. While Leitinger et al. and Othman et al. reported excellent interrater agreement, Goselink et al. ended up with only a moderate interrater agreement and called for “more precise specification of terms such as discharges, fluctuation, and evolution.” Of note, Leitinger et al. already did work to improve definitions in 2015 by applying the ACNS criteria for RDA and fluctuation, leading to fewer false positives and a lesser risk of overtreating static periodic patterns with antiepileptic drugs.^9^ However, in our case, definitions were still not clear enough to obtain a good interrater agreement.

Recently, the ACNS have published new criteria for NCSE that address some of the shortcomings of the Salzburg criteria.^4^ Notably, they introduced a quantified threshold for the amount of seizure activity required to diagnose NCSE: 10 min of a standard recording or 20% of a 60‐min recording. Additionally, they more clearly defined which EEG patterns fall on the ictal‐interictal continuum, excluding the “generalized rhythmic delta activity” pattern, likely due to its nonspecific nature.^21^ Further studies are needed to evaluate whether these adjustments lead to higher diagnostic accuracy.

Our study has some limitations. The patient population is different from the one in the original validation study, including critical ill patients in addition to patients with epilepsy, and mainly short 15–30‐min EEGs. However, we think that it is important to test the criteria in the most realistic setting possible. We cannot exclude that the performances of the Salzburg criteria could have been different in a patient group with a higher level of clinical suspicion. However, in our subanalysis of patients with 4‐h‐long EEG monitorings, the diagnostic accuracy was not higher. Moreover, our laboratory does not routinely administer benzodiazepines or other antiepileptic drugs during the standard EEG and we could thus not evaluate the criterion involving the effect of benzodiazepines or other antiepileptic drugs. However, we think there are several pitfalls to using therapy for diagnostic purposes. In our experience, administration of benzodiazepines are often more confusing than helpful as they are more likely to successfully suppress triphasic waves of an encephalopathy than ictal activity of a NCSE^17^ and the evaluation of the clinical effect may be challenging due to delayed effects, patients falling asleep, and spontaneous fluctuations in others.^22^ Administration of other antiepileptic drugs needs a longer observation time and is more relevant for longer EEG monitorings. However, we think that it is important to be able to apply criteria for NCSE on a first standard EEG, and that these should help in selecting patients for which a longer monitoring is indicated. A last concern regarding the diagnostic interpretation of antiseizure medication effect is that the absence of effect does not exclude the condition: a refractory status epilepticus remains a status epilepticus even if it does not respond to antiseizure medication. Another limitation of our study is that efforts to correct for a possible classification bias may have led to an incorporation bias, which may have overestimated the sensitivity and specificity of the criteria. However, we felt it was more important not to underestimate the sensitivity and specificity of the criteria in this context. Finally, the prevalence of NCSE was lower than estimated when calculating the sample size of the study. Still, the sample size and number of NCSE are larger than for any of the other published studies.

In conclusion, our study contributes to the increasing evidence that the Salzburg EEG criteria lack the specificity needed for systematic implementation in clinical practice. As for now, we argue that diagnosing NCSE relies more on a comprehensive approach than a simple mechanistic algorithm, in a complex interplay between clinical data, pattern recognition and dynamic reading of the EEG. In this setting, criteria such as the Salzburg criteria can still have a role in guiding the EEG reader to salient features of the EEG. To function better on their own, however, the criteria should not only identify EEG patterns that point toward a diagnosis of NCSE, but should also aid more actively in differentiating from this condition – either through general precautionary measures^23^ or preferentially through more specific means. In our opinion, specific improvements should particularly focus on identifying certain seizure patterns with a quantified threshold to qualify for NCSE, and on better specifying the IIC and its “plus”‐features. In addition, they should both describe how to best separate status triphasicus from status epilepticus and should give special attention to epileptic activity in the context of postanoxia. Finally, we support that it is better to avoid the term “possible NCSE” as this may lead to overtreatment and that it is preferable to use the term “IIC.”

## Author Contributions

All authors contributed to the conception and design of the study. L.B.U, L.E, and K.B.N contributed to the acquisition and analysis of data. L.B.U contributed to drafting the text and preparing the figures and tables. All authors reviewed, edited, and approved the final version of the manuscript.

## Conflicts of Interest

None of the authors have any conflicts of interests to report.

## Data Availability

The data supporting this study's findings are available from the corresponding author upon reasonable request.

## References

[1] Gastaut H . Dictionary of Epilepsy, Part 1 Definitions. World Health Organization; 1973.

[2] Trinka E , Cock H , Hesdorffer D , et al. A definition and classification of status epilepticus–report of the ILAE task force on classification of status epilepticus. Epilepsia. 2015;56(10):1515‐1523. 26336950 10.1111/epi.13121

[3] Schomer DL , Lopes da Silva FH . Niedermeyer's Electroencephalography: Basic Principles, Clinical Applications, and Related Fields. 7th ed. Oxford University Press; 2018:543‐545.

[4] Hirsch LJ , Fong MWK , Leitinger M , et al. American clinical neurophysiology Society's standardized critical care EEG terminology: 2021 version. J Clin Neurophysiol. 2021;38(1):1‐29. 33475321 10.1097/WNP.0000000000000806 PMC8135051

[5] Gelisse P , Crespel A , Genton P , Jallon P , Kaplan PW . Lateralized periodic discharges: which patterns are interictal, ictal, or peri‐ictal? Clin Neurophysiol. 2021;132(7):1593‐1603. 34034086 10.1016/j.clinph.2021.04.003

[6] Rossetti AO , Oddo M , Liaudet L , Kaplan PW . Predictors of awakening from postanoxic status epilepticus after therapeutic hypothermia. Neurology. 2009;72(8):744‐749. 19237704 10.1212/01.wnl.0000343006.60851.62

[7] Ruijter BJ , van Putten MJ , Hofmeijer J . Generalized epileptiform discharges in postanoxic encephalopathy: quantitative characterization in relation to outcome. Epilepsia. 2015;56(11):1845‐1854. 26384469 10.1111/epi.13202

[8] Beniczky S , Aurlien H , Brogger JC , et al. Standardized computer‐based organized reporting of EEG: SCORE. Epilepsia. 2013;54(6):1112‐1124. 23506075 10.1111/epi.12135 PMC3759702

[9] Leitinger M , Beniczky S , Rohracher A , et al. Salzburg consensus criteria for non‐convulsive status epilepticus–approach to clinical application. Epilepsy Behav. 2015;49:158‐163. 26092326 10.1016/j.yebeh.2015.05.007

[10] Leitinger M , Trinka E , Gardella E , et al. Diagnostic accuracy of the Salzburg EEG criteria for non‐convulsive status epilepticus: a retrospective study. Lancet Neurol. 2016;15(10):1054‐1062. 27571157 10.1016/S1474-4422(16)30137-5

[11] Barrett BJ , Fardy JM . Evaluation of diagnostic tests. Methods Mol Biol. 2021;2249:319‐333. 33871852 10.1007/978-1-0716-1138-8_18

[12] Cohen JF , Korevaar DA , Altman DG , et al. STARD 2015 guidelines for reporting diagnostic accuracy studies: explanation and elaboration. BMJ Open. 2016;6(11):e012799. 10.1136/bmjopen-2016-012799 PMC5128957 28137831

[13] Buderer NM . Statistical methodology: I. Incorporating the prevalence of disease into the sample size calculation for sensitivity and specificity. Acad Emerg Med. 1996;3(9):895‐900. 8870764 10.1111/j.1553-2712.1996.tb03538.x

[14] Goselink RJM , van Dillen JJ , Aerts M , et al. The difficulty of diagnosing NCSE in clinical practice; external validation of the Salzburg criteria. Epilepsia. 2019;60(8):e88‐e92. 31318040 10.1111/epi.16289 PMC6852511

[15] Othman AS , Meletti S , Giovannini G . The EEG diagnosis of NCSE: concordance between clinical practice and Salzburg criteria for NCSE. Seizure. 2020;79:1‐7. 32371363 10.1016/j.seizure.2020.04.010

[16] Krogstad MH , Hogenhaven H , Beier CP , Kroigard T . Nonconvulsive status epilepticus: validating the Salzburg criteria against an expert EEG examiner. J Clin Neurophysiol. 2019;36(2):141‐145. 30585889 10.1097/WNP.0000000000000556

[17] Bicchi MM , Alkhachroum A , Kanner AM . Status triphasicus versus status epilepticus? J Clin Neurophysiol. 2021;38(5):376‐383. 34155181 10.1097/WNP.0000000000000764

[18] Drislane FW , Blum AS , Schomer DL . Focal status epilepticus: clinical features and significance of different EEG patterns. Epilepsia. 1999;40(9):1254‐1260. 10487189 10.1111/j.1528-1157.1999.tb00855.x

[19] Treiman DM , Walton NY , Kendrick C . A progressive sequence of electroencephalographic changes during generalized convulsive status epilepticus. Epilepsy Res. 1990;5(1):49‐60. 2303022 10.1016/0920-1211(90)90065-4

[20] Suzette LaRoche VR . The ictal‐Interictal continuum. In: LaRoche S , Haider H , eds. Handbook of ICU EEG Monitoring; 2nd ed. Springer Publishing Co Inc; 2018:193‐197.

[21] Rodriguez Ruiz A , Vlachy J , Lee JW , et al. Association of periodic and rhythmic electroencephalographic patterns with seizures in critically ill patients. JAMA Neurol. 2017;74(2):181‐188. 27992625 10.1001/jamaneurol.2016.4990

[22] Fernandez‐Torre JL . Nonconvulsive status epilepticus versus triphasic encephalopathy. Epilepsia. 2007;48(5):1035 author reply 6. 17509011 10.1111/j.1528-1167.2007.01009_7.x

[23] Maulsby RL . Some guidelines for assessment of spikes and sharp waves in EEG tracings. Am J EEG Technol. 1971;11(1):3‐16.

